# Behavior and exocrine glands in the myrmecophilous beetle *Dinarda dentata* (Gravenhorst, 1806) (Coleoptera: Staphylinidae: Aleocharinae)

**DOI:** 10.1371/journal.pone.0210524

**Published:** 2019-01-11

**Authors:** Bert Hölldobler, Christina L. Kwapich

**Affiliations:** 1 Social Insect Research Group, School of Life Sciences, Arizona State University, Tempe, Arizona, United States of America; 2 Biozentrum, Zoology II, University of Würzburg, Bavaria, Germany; University of Arizona, UNITED STATES

## Abstract

The nests of advanced eusocial ant species can be considered ecological islands with a diversity of ecological niches inhabited by not only the ants and their brood, but also a multitude of other organisms adapted to particular niches. In the current paper, we describe the myrmecophilous behavior and the exocrine glands that enable the staphylinid beetle *Dinarda dentata* to live closely with its host ants *Formica sanguinea*. We confirm previous anecdotal descriptions of the beetle’s ability to snatch regurgitated food from ants that arrive with a full crop in the peripheral nest chambers, and describe how the beetle is able to appease its host ants and dull initial aggression in the ants.

## Introduction

The complex nest structures of many highly eusocial ant species and the immediate surroundings constitute micro-ecosystems in which the resident ants coexist with numerous co-inhabitants. Indeed, the nest area of complex, evolutionarily derived ant societies can be subdivided into functional sites, such as brood chambers, peripheral nest chambers, middens and foraging routes. Co-inhabitants include a large variety of beetles, wasps, flies, mites and other arthropods [1–3] that are specially adapted to make their living inside specific niches created by the ants. Some of these myrmecophiles (organisms that live in close association with ants) are commensals, others are predators or kleptoparasites. Although some rather unspecialized myrmecophiles succeed in living in the core of the nest without receiving food or grooming by their ant hosts[4], the most intriguing examples live inside the colony’s brood chambers, where they prey on the ant brood and in addition, are not only tolerated, but also tended and fed by the ants. Examples of the highly adapted parasitic symbionts are the rove beetles of the genera *Lomechusoides* (formerly called *Lomechusa*) and *Lomechusa* (formerly called *Atemeles*)[5]. Such highly specialized myrmecophiles exhibit a multitude of morphological, chemical, and behavioral adaptations that make them not only accepted by, but also in many ways even privileged within the host colony [6–10]. One can say that during their evolution, these myrmecophiles have succeeded in breaking parts of their hosts’ communication code, which enables them to exploit the social provision networks inside the ant colony. This extreme mode of myrmecophily must be considered a much-derived evolutionary grade of myrmecophilous parasitism in rove beetles.

In order to conceive of possible evolutionary pathways leading to such astounding myrmecophilous adaptations, we have previously investigated other rove beetle species of the genus *Pella*, that like *Lomechusoides* and *Lomechusa*, belong to the subfamily Aleocharinae, but live as scavengers and predators in the peripheral zones around the nest, at the “garbage dumps,” and on the ants’ foraging trunk routes [10]. Some of the behavioral features of *Pella* seem to closely resemble those of *Lomechusa* and *Lomechusoides*, such as employing a chemical appeasement behavior when encountering host ants [9]. This appeasement behavior appears to be an important prerequisite for living closely with ants. Indeed, our observations indicate that *Pella* only rarely employs strong smelling tergal gland secretions when they are near the host ant colony. Similar results were obtained with *Lomechusa* and *Lomechusoides* [8, 9]. Although *Pella* species are endowed with the appeasement gland complex (see also [11, 12]), they entirely lack the specific adoption glands described in *Lomechusa* and *Lomechusoides*. In fact, this genus does not penetrate to the internal chambers of the host colony’s nest. Instead, it mainly eats dead ants deposited in the middens or hunts ants on the foraging trail and near the nest entrance, especially injured individuals [10].

The aleocharine species *Dinarda dentata* appears to represent an evolutionary grade between *Pella* on one side, and *Lomechusa* and *Lomechusoides*, on the other side. It mainly dwells in the peripheral chambers of the nests of its host ants (*Formica sanguinea*). However, it has never been seen hunting host ants. Instead, Wasmann [6, 13] reports that beetles eat debris discarded by the host ants and are occasionally seen inserting themselves between two food exchanging ants, snatching a food droplet that was about to be passed from one ant to another. He also reported one incidence of having observed a *Dinarda* beetle with an ant egg between its mandibles. In a more recent paper on the con-generic *Dinarda maerkelii*, Parmentier et al. [14] reported occasional egg and larvae predation, and presented evidence for trophallaxis with the host ants *Formica polyctena* and *F*. *rufa*. They also provided quantitative observational data on within-nest distribution of the beetles and acceptance of the beetles by the host ants. Wasmann also suggests that the *Dinarda* beetles may keep the host ants free from mite infections, but this statement is solely based on circumstantial observations [13].

Except for these observations and some sporadic indications concerning the exocrine glands of *Dinarda* [15, 16], no analysis of the behavioral mechanisms that enable Dinarda dentata beetles to coexist with host ants nor in-depth analysis of the glandular endowment of this myrmecophilous beetle genus has been published. The current paper will contribute to a better understanding of the myrmecophilous adaptations of *Dinarda dentata*.

## Materials and methods

Most of the data in this paper were collected in years 1968, 1969 and 1972. *Dinarda dentata* beetles were collected in the xerothermic limestone habitats of the lower Franconia surroundings of the river Main (Germany), from nests of the ant *Formica sanguinea*. In the laboratory, the beetles were housed in formicaria together with colonies of their host species, *F*. *sanguinea* (which in some cases also contained workers of the so-called slave species *F*. *fusca*). The formicaria were constructed out of plaster of Paris casts and included 16 chambers each measuring 4 X 4 cm. Each plaster nest was covered by a glass plate and placed inside a Plexiglas box (26 x 20 cm) as described by Hölldobler et al. [9]. In the Plexiglas arena, honey-sucrose water, chopped cockroaches (*Blatta germanica*) and mealworm larvae (*Tenibrio molitor*) were provided as food. The *Dinarda* were kept in five separate colonies; sometimes they were housed together with *Lomechusoides* beetles in the ant nests.

Freshly collected beetles were released into Plexiglas arenas and the behavioral interactions between ants and *Dinarda* were observed. Approximately one week after introduction, we took irregular snapshot counts of the beetles’ location in the nest across a three-week period (n = 5 colonies, 10 counts per colony, 41 beetles total). For comparison we also counted the locations of the myrmecophilous beetle *Lomechusoides strumosus* in separate *Formica sanguinea* colonies (n = 12 colonies, 9–12 counts per colony over a period 3 to 4 weeks, 115 beetles total).

To determine whether the beetles preferably sojourn in the brood chambers of the ant nest, beetle abundance in brood chambers and non-brood chambers was compared. For each colony, we averaged the number of beetles in each chamber type across samples dates. These independent averages were then summed within species. The maximum number of brood chambers per sample was also averaged within colonies and pooled by species. Beetles found in the arena represented a low proportion of the total (less than 5% on average), and were excluded from analysis so that only the spatial distribution within the nest was considered for statistical analysis. Brood occupied a maximum of 3 out of 16 chambers in nests containing *D*. *dentata*, and 4–9 of 16 chambers in nests containing *L*. *strumosus*. Preferences for brood were determined using two-sided exact goodness of fit tests that compared the observed distribution of beetles across brood chambers to the expected distribution for each species.

To test how much *Dinarda* beetles were able to siphon off regurgitated food from their host workers, ants were fed honey-sucrose water labeled with the radioisotope ^32^P, which was added as orthophosphate at a specific activity of 1–5 μc/mL. The liquid food was offered to the ants in small glass dishes. After feeding was completed, the ants were bathed in distilled water to decontaminate them. The ants were then placed in petri dishes to dry, the floor of which was covered with absorbent cellulose paper on which ants could walk around. After about 30 minutes, the ants were taken out. After each such maneuver, the petri dish was cleaned, and new cellulose paper was put in. This procedure was first developed by Werner Kloft [17] and tested successfully in many subsequent tracer experiments with ants.

To determine if beetles exploited host ant trophallaxis, we conducted two series feeding tests with *Dinarda* beetles. Series (A) consisted of three test groups, each containing ten host ants fed with radioactive honey water, and five *Dinarda* beetles taken from the same source colony. In Series B, five workers from four test groups were starved over an 8-day period. The starved workers were then fed with ^32^P labeled honey-water and placed with 10 nest mates and 5 *Dinarda* beetles. We predicted that the ants in series B would engage in more trophallaxis acts, increasing the opportunity for beetles to steal food.

One day later, we measured each individual ant and beetles of each of the four groups. For measurements, we used a liquid scintillation counter with an automatic sample changer (Philips, Eindhoven). However, we did not use liquid scintillation but instead placed individual live ants or beetles into the dry vials and measured the quantity of food by using the impulses per 100 seconds caused by Cherenkov radiation.

For histological investigations, specimens were fixed in alcoholic Bouin (Dubosq Brasil) or Carnoy [18], embedded in methyl-methacrylate, and sectioned 5 μm thick with a Jung Tetrander microtome [19]. The staining was Hematoxylin-Eosin or Heidenhain Azan. All histological preparations, the radioactive tracer experiment, and the behavioral studies were conducted at the laboratory of B. Hölldobler at the University of Frankfurt. Re-analysis of the histological sections, SEM work, and the entire data analysis were carried out at the School of Life Sciences at Arizona State University.

## Results

### Exocrine glands

**Fig 1:**
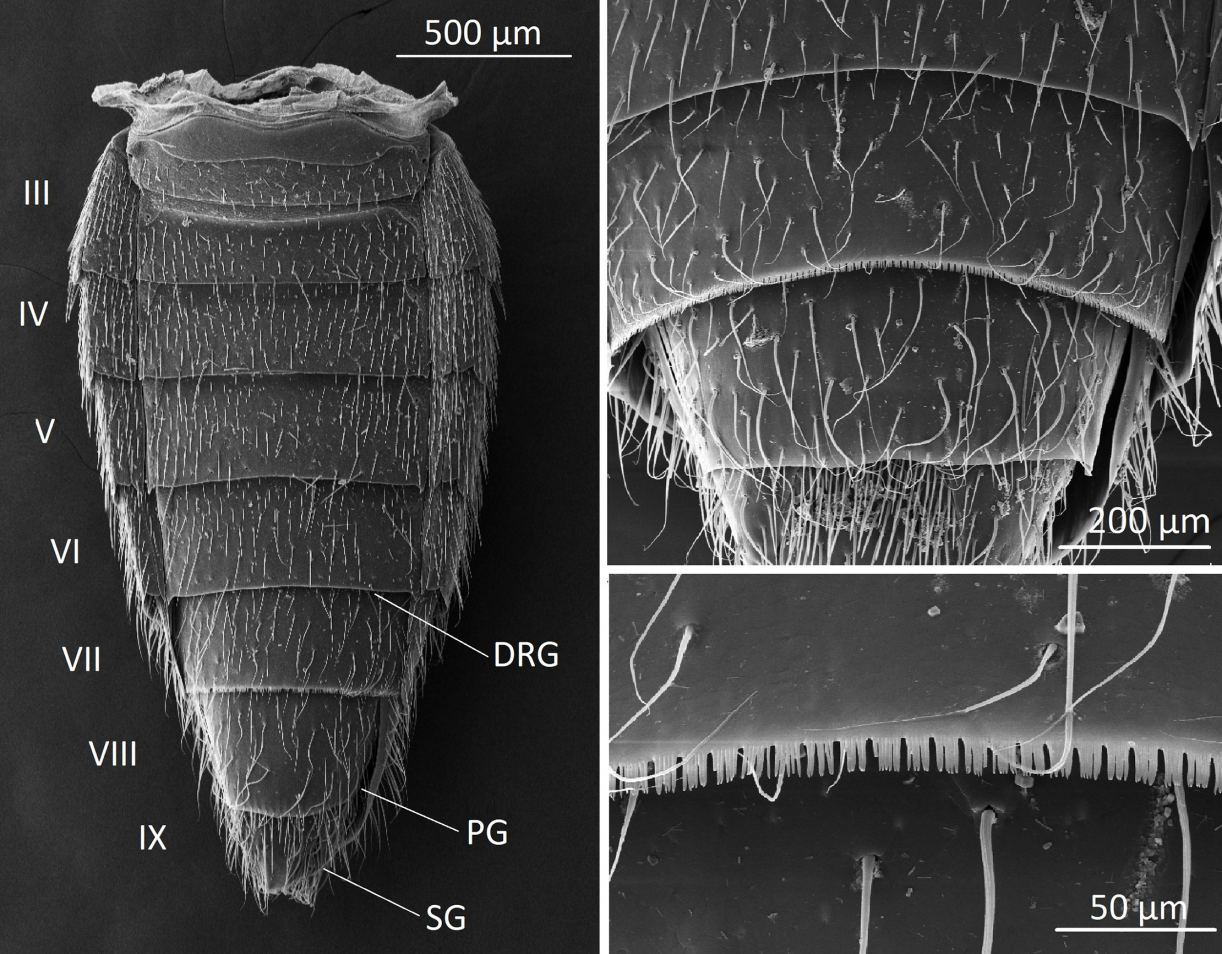
Scanning electron microscopic images of the abdomen of *Dinarda dentata*. a) Whole abdomen of the beetle showing the externally visible 8 full ring (tergum plus sternum) segments, beginning with segment III to IX. The repellent-defense tergal gland opens between segments VI and VII tergites. The paired pleural gland opens dorsa-laterally between the segment VIII and IX. The paired sternal gland opens at the posterior–lateral sides of segment IX. b) The posterior rim of tergite VII features a serrate structure not present in the other tergites or sternites. c) Close-up of the serrate structure along the posterior rim of tergite VII.

**Fig 2:**
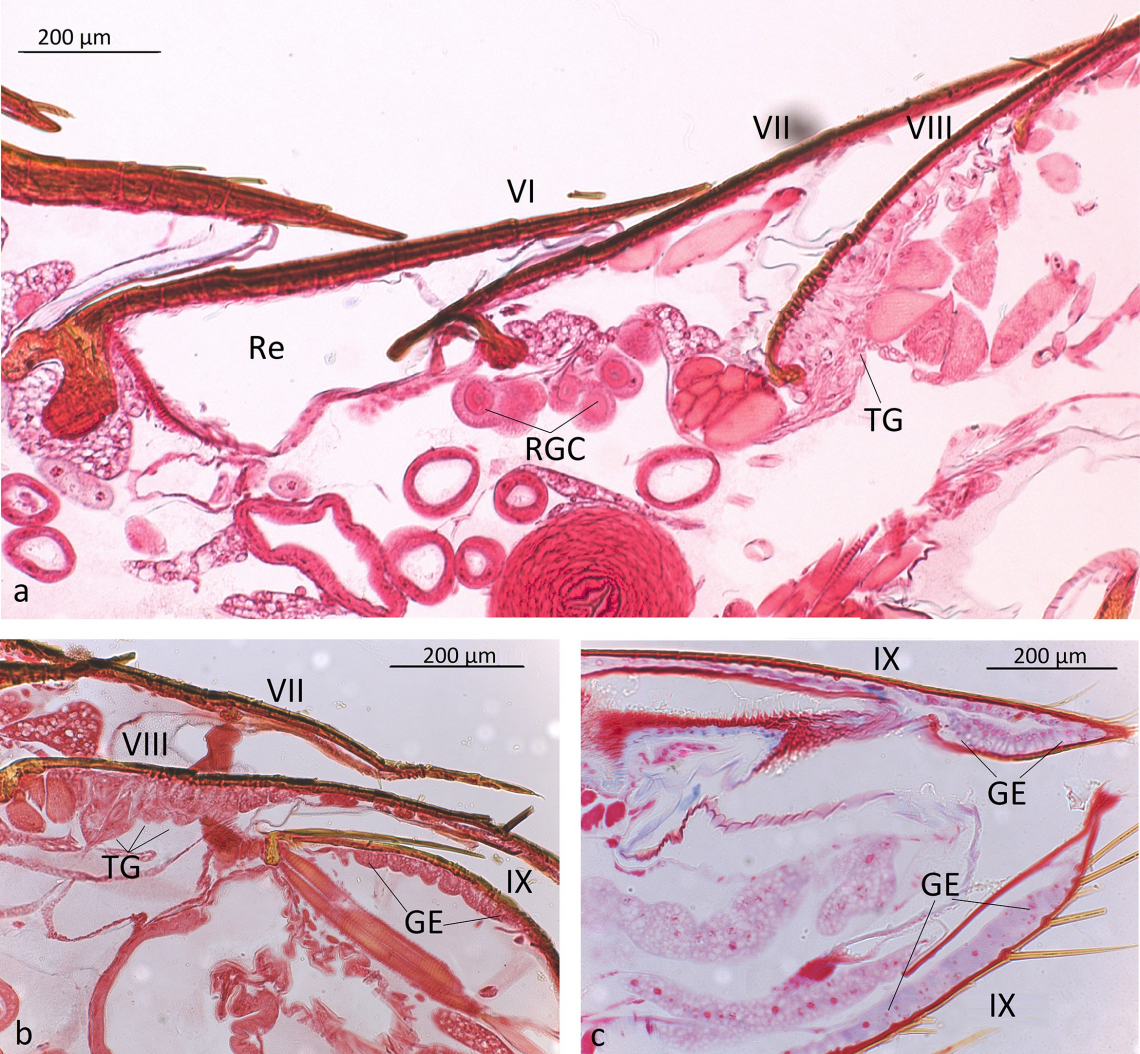
Longitudinal sections through the abdominal posterior segments of *Dinarda dentata*. a) Section through 6^th^, 7^th^ and 8^th^ tergites, showing the repellent-defense tergal gland between 6^th^ and 7^th^ tergites. Re, reservoir of the repellent defense gland; RGC repellent gland cells; TG, parts of the tergal gland in the 8^th^ tergite. b) Sections through 7^th^, 8^th^, and 9^th^ tergites, showing the tergal glandular epithelium (GE) in the 8^th^ tergite and c) in the 9^th^ tergite and sternite.

In staphylinid beetles, the first fully developed abdominal segmental ring (tergite plus sternite) is usually considered to be the 3^rd^ abdominal segment [20]. This is also the case in *Dinarda dentata* beetles (Fig 1A). The most obvious abdominal gland structure is the tergal gland between tergites VI and VII. It was first discovered by Jordan [15]in *Lomechusa* and *Lomechusoides*, as well as in *Dinarda* and later confirmed by Pasteels [16], and Hölldobler [8] and Hölldobler et al. [9]. It consists of paired clusters of glandular cells with duct cells that open into paired pouches that are formed by invagination of the intersegmental membrane between the 6^th^ and 7^th^ tergites. In contrast to *Lomechusoides* and *Lomechusa*, *Dinarda* lacks the massive glandular epithelium located in the reservoir membrane (Fig 2A). Jordan identified this gland correctly as a defensive gland, which produces a repellent secretion. Interestingly, the posterior edge of the 7^th^ tergite features a comb-like or serrated fringe which appears much more pronounced in *Dinarda* than in *Lomechusa* and *Lomechusoides* (Fig 1A and 1C). Perhaps this structure is involved in the dispersion of secretion from the tergal gland in the 8^th^ tergite, the openings of which are covered by the preceding 7^th^ tergite. This gland is absent in *Lomechusoides* and *Lomechusa*.

**Fig 3:**
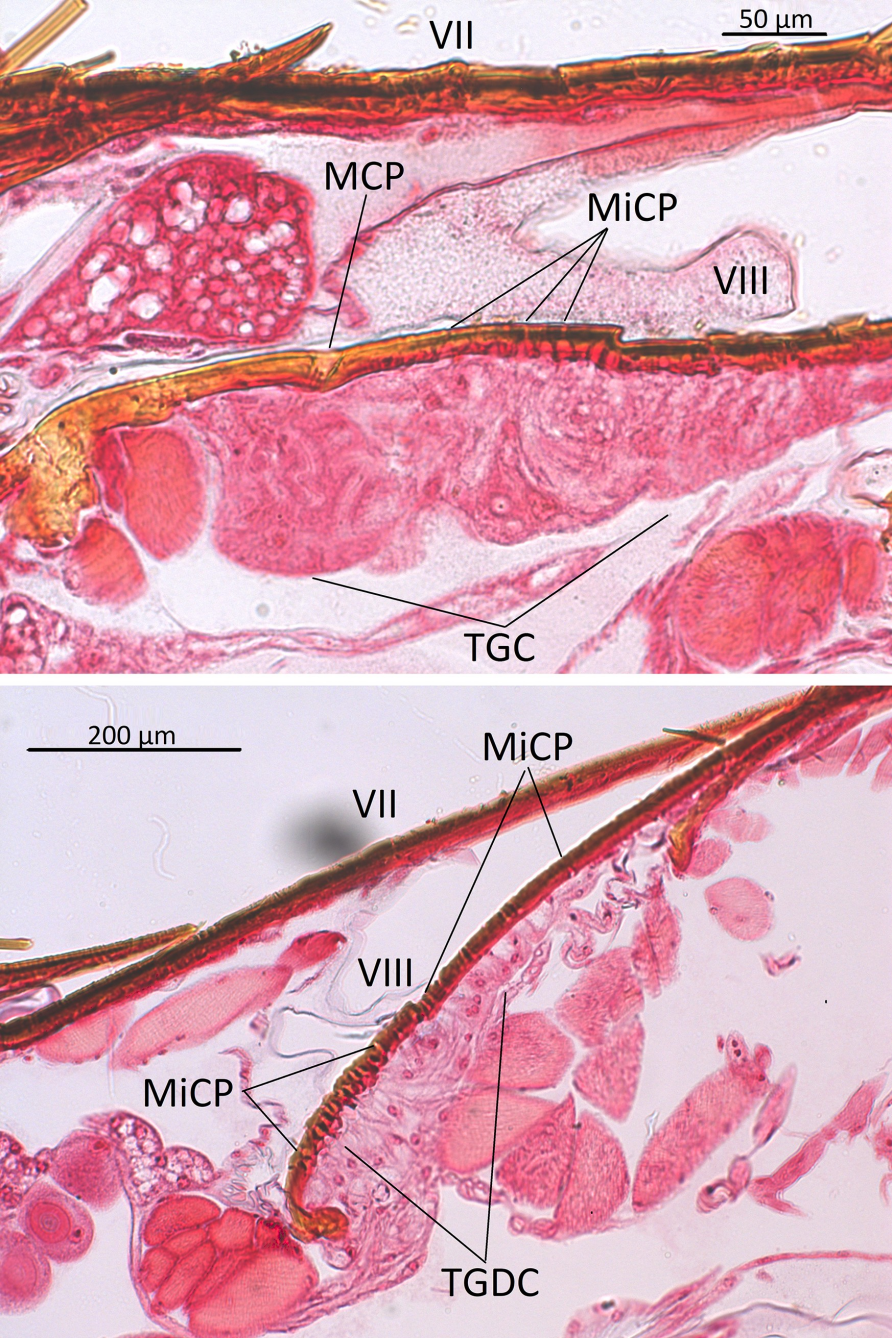
Longitudinal sections through the tergal gland in segment VIII of *Dinarda dentata*. Above: Section through the median zone of the tergal gland. TGC, tergal gland cells; MCP, major cuticle pore; MiCP, minor cuticle pores. Below: Section through lateral zone of the tergal gland. Densely packed ducts of duct cells that open through the MiCPs, and cells with vacuoles, TGDC.

**Fig 4:**
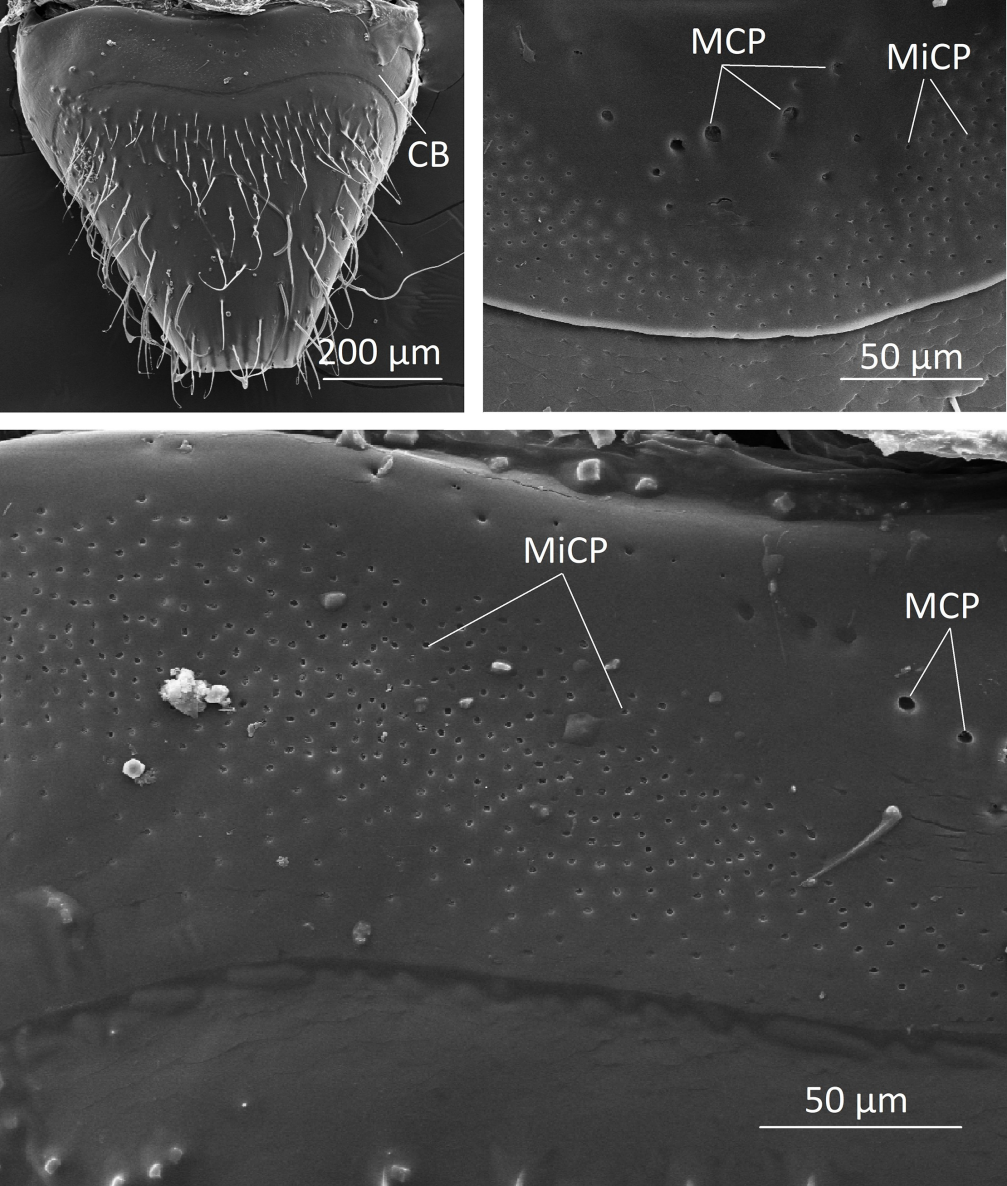
Scanning electron microscopic images of the 8^th^ abdominal tergite of *Dinarda dentata*. a) Whole view of tergite VII. CB, cuticle belt of tergal gland; MiCP, minor cuticle pores; MCP, major cuticlepores. b) Close-up of cuticle belt with minor cuticlepores mainly in the lateral regions and major cuticle pores in the central region. c) Left portion the cuticle belt that shows mini-pores only in the lateral region, macro-pores in the central region.

**Fig 5:**
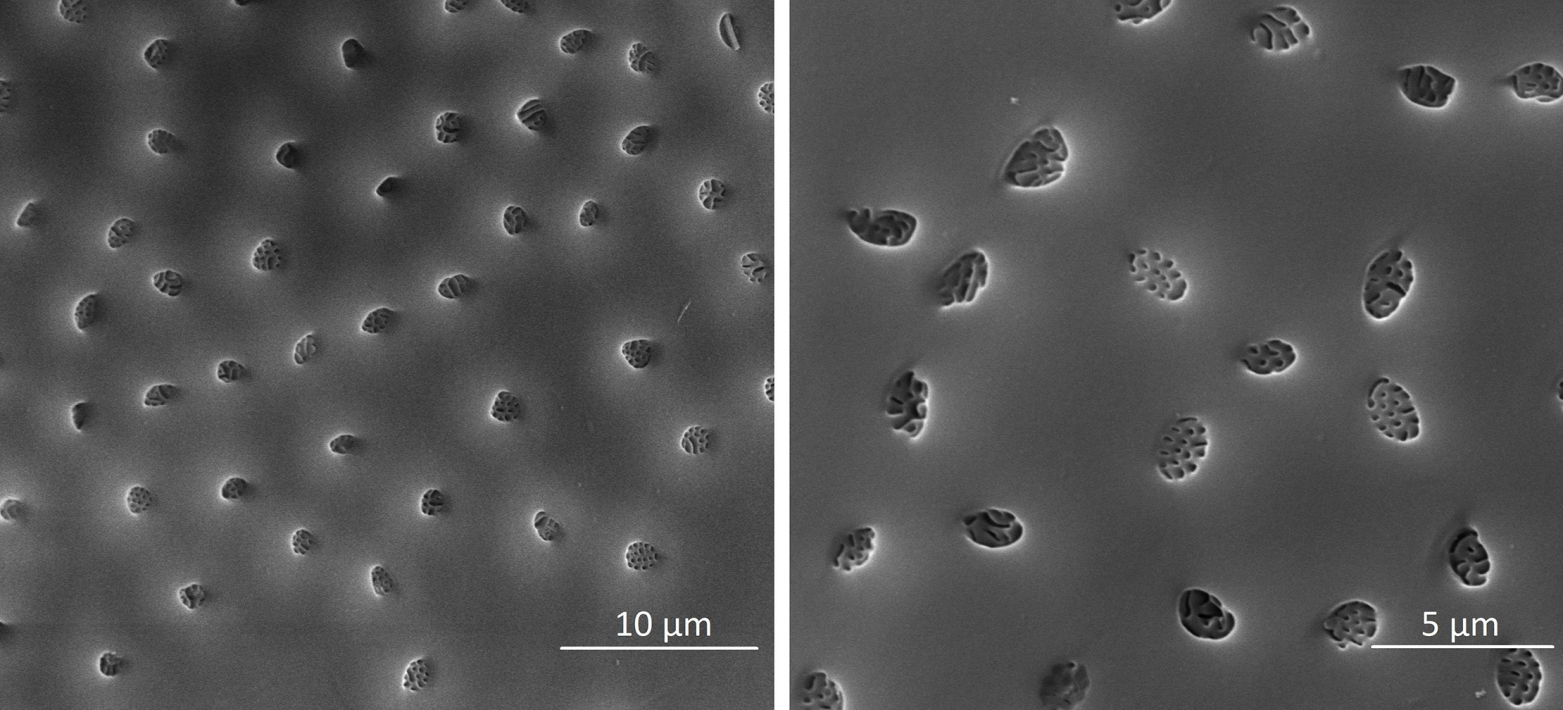
Scanning electron microscopic images of the minor cuticlepores in the cuticle belt of the tergal gland in 8^th^ abdominal tergite.

**Fig 6:**
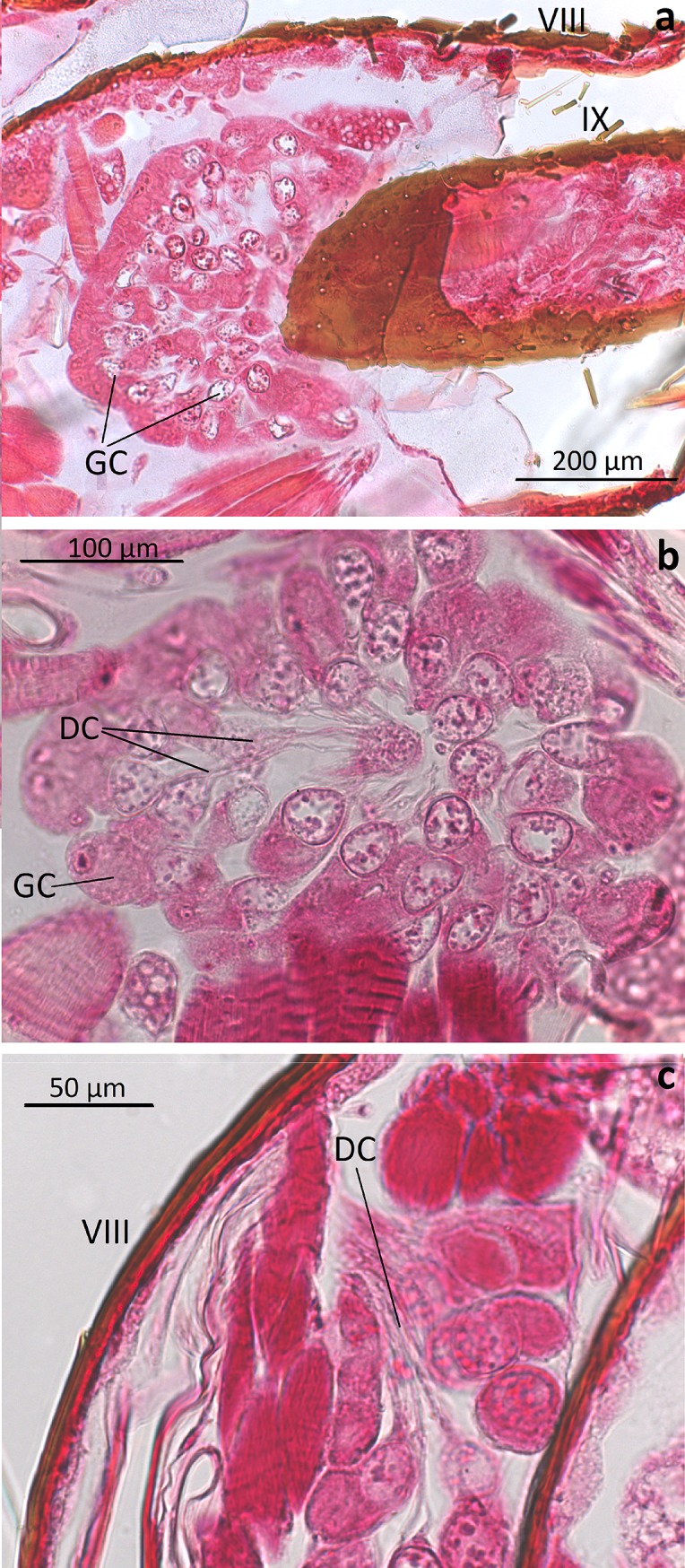
Sections through the pleural gland in the 8^th^ abdominal segment of *Dinarda dentata*. a) Pleural gland located between 8^th^ and 9^th^ abdominal segment. GC, glandular cells. b) Close-up of pleural gland with glandular cell (GC) and duct cells (DC). c) Transversal section through pleural gland, showing glandular cells (GC) and duct cell (DC) opening dorsa-laterally between the 8^th^ and 9^th^ abdominal segments.

**Fig 7:**
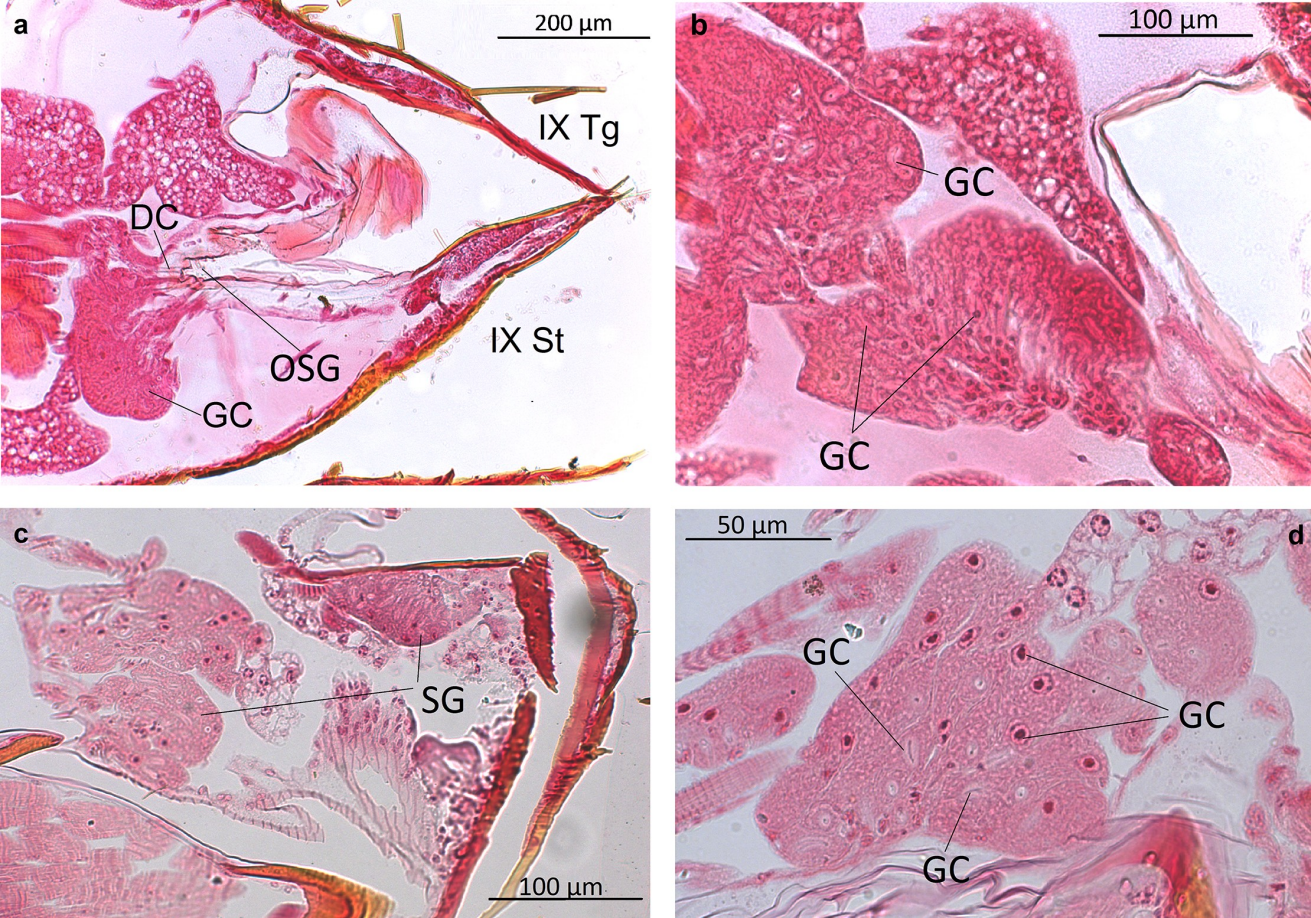
Longitudinal sections through the 9^th^ abdominal segment of *Dinarda dentata*. a) Tip of the 9^th^ segment showing the sternal gland of a *Dinarda* female. Tg, tergite; St, sternite; OSG, opening of the sternal gland; GC, glandular cell; DC, duct cell. b) Magnification of the sternal gland in *Dinarda* female. GC, glandular cell; DC, duct cell. c) Sternal land of a *Dinarda* male. SG, sternal gland. d) Magnification of the sternal gland in a *Dinarda* male. GC, glandular cells.

**Fig 8:**
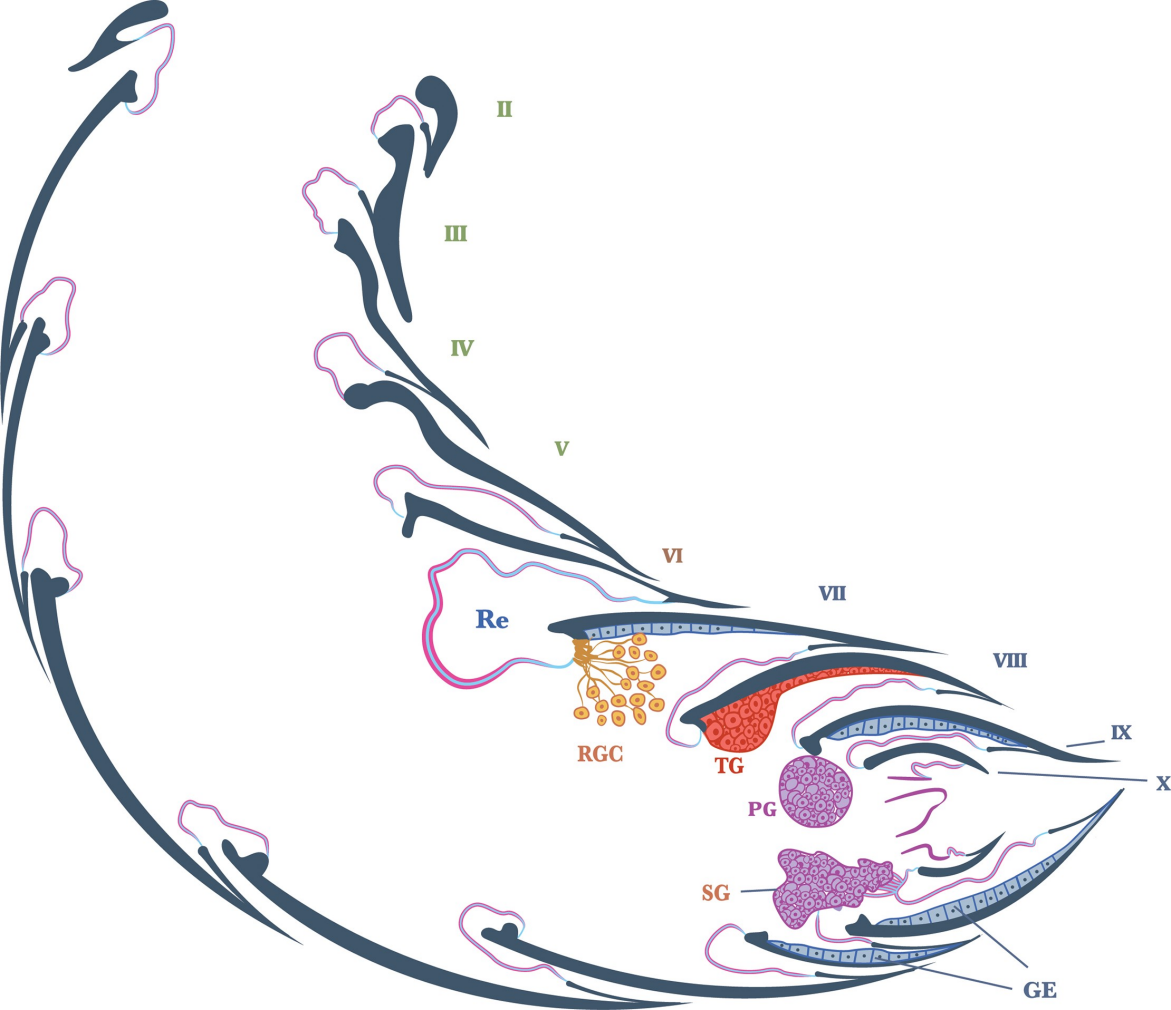
Schematic illustration of the exocrine glands in the abdominal tip of *Dinarda dentata*. RGC, defense repellent gland cells; Re, reservoir of dense repellent gland; TG, tergal gland in 8^th^ abdominal tergite; PG, pleural gland; SG, sternal gland; GE, glandular epithelium.

*Dinarda* beetles do not have the so-called adoption glands found in several anterior paratergites of *Lomechusa* and *Lomechusoides* [8, 9, 15]. We did not find specific secretory hypodermal cells openings through the cuticle in these regions in *D*. *dentata* beetles. We were unable to confirm Jordan’s [15] claim that simple hypodermal glandular cells associated with innervated setae are present in the front paratergites. We did, however, find a well-developed glandular epithelium in the 8^th^ and 9^th^ tergites and sternites (Fig 2B and 2C) and some hypodermal glandular cells in the anterior section of the 7^th^ tergite. The glandular epithelium in the anterior tergal zone of the 8^th^ abdominal segment is particularly well developed (Fig 3B and 3C) in the central part of the 8^th^ segment where the duct cells of the densely packed glandular cells open through the major cuticle pores shown in Fig 3A and Fig 4. The lateral parts of this tergal gland are associated with a “sponge-like” modification of the epidermis (Fig 3B) consisting of vacuoles and bundles of ducts that open through the many minor cuticle pore openings (Fig 3B). Scanning electron microscopic images of the cuticle surface of this region revealed the densely packed minor cuticle pores and the much fewer major cuticle pores in the median zone of the anterior tergite VIII (Fig 5). Pasteels [16] mentions a gland in the 8^th^ tergite of *Dinarda* but his description does not match the kind of gland we found. Pasteels did not illustrate this gland and in fact, it might be the same gland we found, because no other gland in this region could be detected. Between the 8^th^ and 9^th^ segments we found paired clusters of glandular cells, the duct cells of which appear to open in the pleural area through the intersegmental membrane between the 8^th^ and 9^th^ abdominal segments (Fig 6). We assume these glands are identical to those Pasteels [16] calls post-pleural glands, which, according to Pasteels, open between the 7^th^ and 8^th^ segments. Our histological pictures suggest that the openings are more likely between the 8^th^ and 9^th^ dorsal pleural segments. Finally, we discovered relatively large sternal gland consisting of paired clusters of many glandular cells and duct cells that open in the area of 9^th^ sternite (Fig 7). We found it in females and males, although in females the glandular clusters appear to be larger. Interestingly, neither Jordan [15] nor Pasteels [16] mentioned this gland. We previously detected similar glands in several species of the genus *Pella* [10] and it is possible that the large paired clusters of glandular cells which open in the pleura-sternal region of the 9^th^ abdominal segment in *Lomechusoides* and *Lomechusa*, which we called pleural glands, are in fact homologous with the sternal glands found in Dinarda and Pella. We also suspect that the large glandular cells with internal reservoirs in the posterior part of the abdomen of *Lomechusoides* and *Lomechusa* [9], of which we were unable to determine the openings of the duct cells, belong to the gland which Pasteels [16] called post-pleural gland. Fig 8 schematically summarizes the location of the various glands found in the abdominal tip of *Dinarda dentata*.

### Behavior

**Fig 9:**
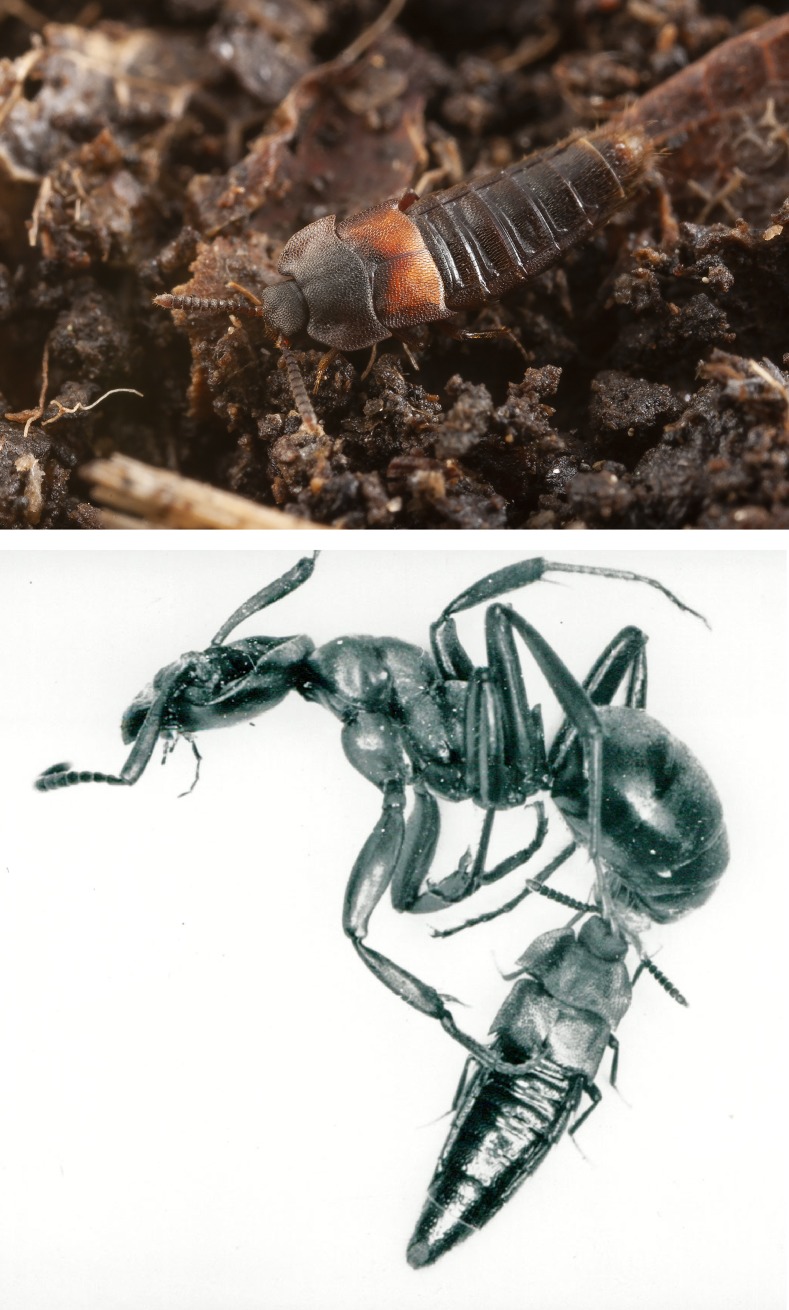
The scavenger *Dinarda dentata*. Above: *Dinarda dentata* beetle in the midden of a natural ant nest. Photo courtesy Pavel Krásenský. Below: *Dinarda dentata* beetle feeding on a dead host ant *Formica sanguinea*.

**Fig 10:**
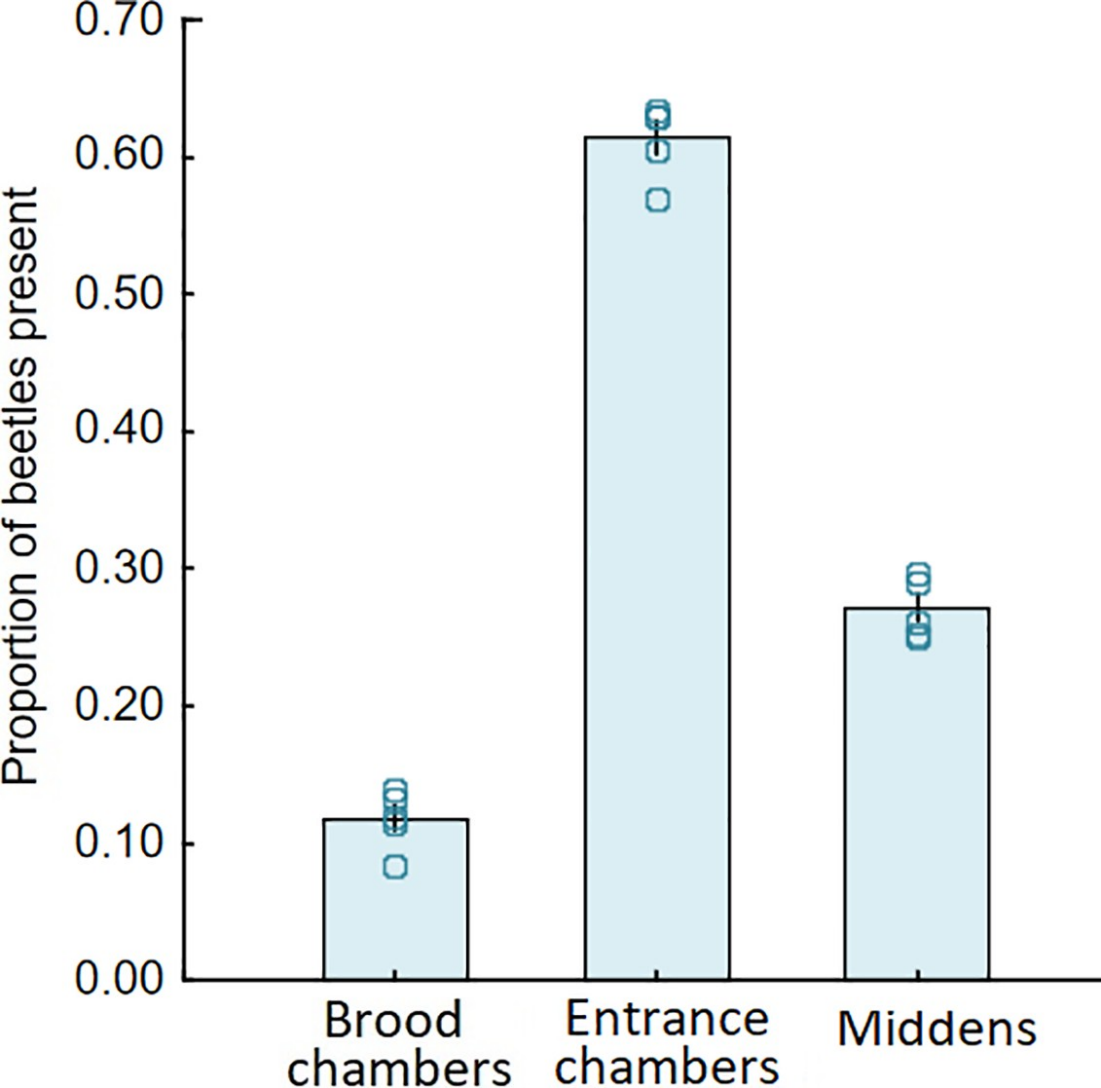
Location of *Dinarda* in nest. The mean proportion of beetles present in three nest locations, across five colony averages (bars represent the standard error of the mean). Less than 12% of beetles were found in brood chambers. Most beetles were found inside entrance chambers (mean proportion = 0.61, SD 0.03), followed by middens (0.27, SD 0.02).

When beetles from the field were introduced into laboratory colonies, we noticed that they tended to avoid contact with the ants. In most cases, the beetles remained outside the arena for several days, often staying near the external midden in the arena, where they foraged on discarded debris such as dead ants or leftover pieces of prey (Fig 9). Although this behavior could last several days, we observed brief entries into the peripheral nest chambers near the nest exit. The ants, when making antennal contact with a beetle, exhibited slightly aggressive behavior with gaping mandibles and forward jerks. In most cases, the beetle rapidly bent the anterior part of its abdomen upwards and escaped skillfully. Usually it took several days for the beetle to settle inside of the nest, and for the ants to reduce their aggressive behavior toward the beetle. In a survey over three weeks, we recorded the beetle occupying the peripheral nest chambers 61% of the time. Beetles occasionally appeared in the internal kitchen midden and the exterior midden in the arena. Unlike *Lomechusa*, and *Lomechusoides*, which shows a significant preference for brood chambers (Table 1), *Dinarda* were less frequently found in the brood chambers. *D*. *dentata* appeared in brood chambers in less than 15% of within-nest observations, and 12% of whole arena observations (Table 1; Fig 10). These findings match those reported by Parmentier et. al. [4] for the congeneric species *D*. *maerkelii*.

**Table 1:** Results of two exact goodness of fit tests examining differences in *D*. *dentata* and *L*. *strumosus* preferences for brood chambers within nests.

Species	No. Colonies	Chamber Type	Chamber Number	Total Beetle No. In Nest	Obs. Beetle No.	Exp. Beetle No.	Obs. Prop.	Exp. Prob.	Exact goodness of fit p-value^a^	95% conf. interval
***D*. *dent*.**	5	Brood	15/80	37	5	7	0.14	0.19	0.53	0.045–0.28
***D*. *dent*.**		Other	65/80		32	30	0.86	0.81		
***L*. *strum*.**	12	Brood	79/192	103	97	42	0.94	0.41	< 0.0001*	0.88–0.98
***L*. *strum*.**		Other	113/192		6	61	0.06	0.59		

^a^significance = α < 0.05, denoted by an asterisks

**Fig 11:**
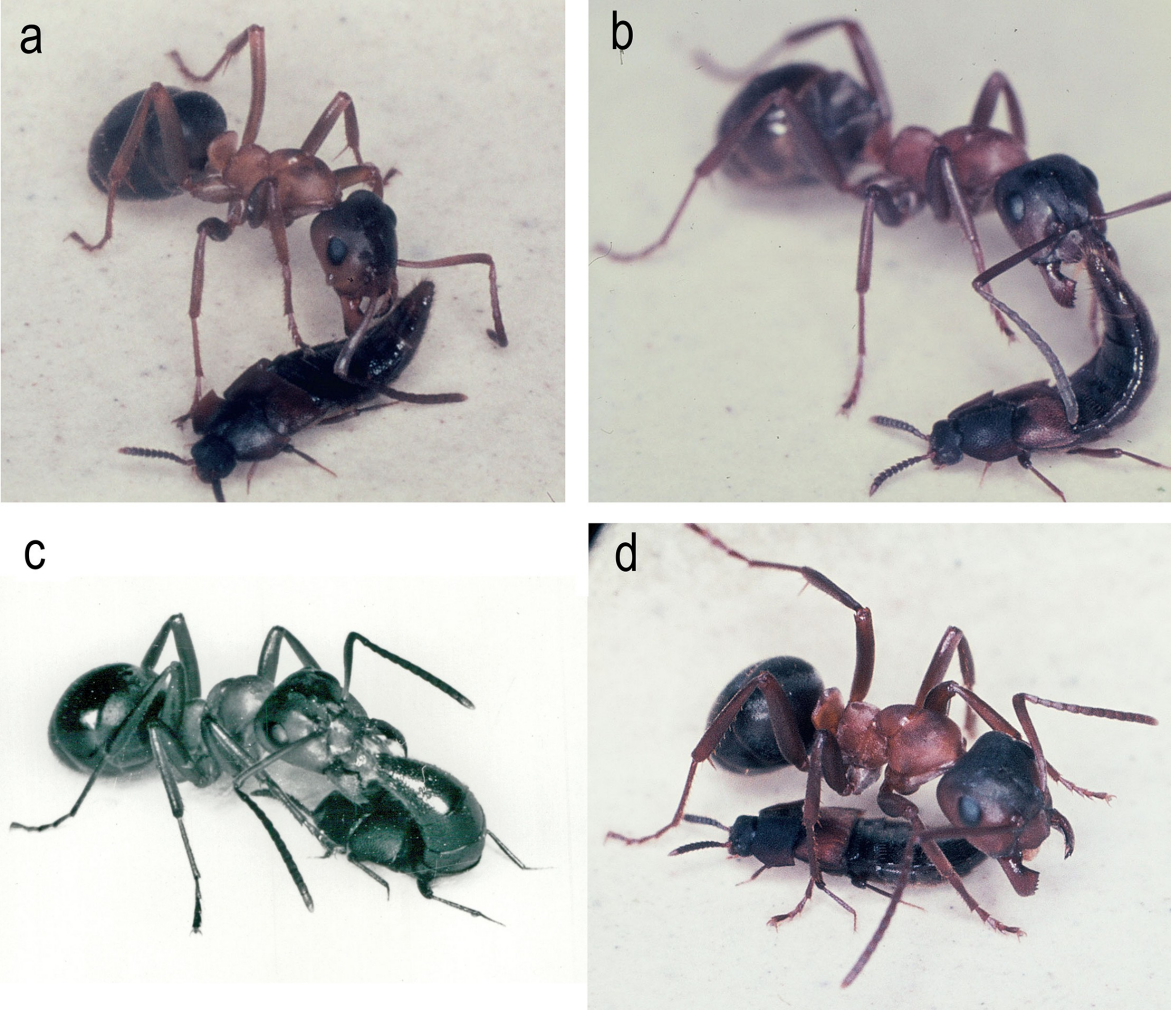
Appeasement interactions between *Dinarda dentata* and host ant *Formica sanguinea*. a) The host ant encounters a *Dinarda* beetle. The beetle reacts by raising its abdomen. b) The beetle’s abdomen is raised to reach the ant’s mouthparts. c) The ant extrudes its lower lip (labium) and licks the liquid at the abdominal tip of the beetle. d) This appeases the ant’s aggressive intentions and the beetle swiftly escapes.

Although *Dinarda* does not keep its abdomen permanently curved upwards, as *Lomechusa* and *Lomechusoides* do, whenever it comes close to its host ants, it bends the abdomen upward, pointing the tip of the abdomen towards the ant’s head (Fig 11). Previous observers interpreted this behavior as an intention movement, possibly leading to the discharge of the repellent secretion from the defense tergal gland [6, 15]. We observed only a few instances where field caught *Dinarda* beetles apparently discharged the repellent secretion when introduced to foreign host ants and were attacked by some ants. The ants exhibited a brief repellent reaction moving head and thorax rapidly sideways or displaying other aversive behavior patterns. In all cases, the beetles successfully escaped. Wasmann [6] however, reports some instances where the *Dinarda* beetle was killed by host ants.

*Dinarda* beetles regularly lift the abdominal tip upwards when contacted by an ant, but in most cases, the repellent secretion from the tergal gland is not ejected. Instead, the appeasement gland complex located in the abdominal tip is presented to the ants (Fig 11). In most cases, this stops aggression in the ants, which lick the beetle’s abdominal tip, where occasionally one can notice an opaque liquid. We do not know whether this liquid originates from the pleural gland, the sternal gland or from both. It is possible that sporadically, the hindgut contents are also discharged by the beetle during the appeasement process. In any case, this appeasement interaction between beetle and ants is very effective and enables the beetles to avoid ant aggression.

**Fig 12:**
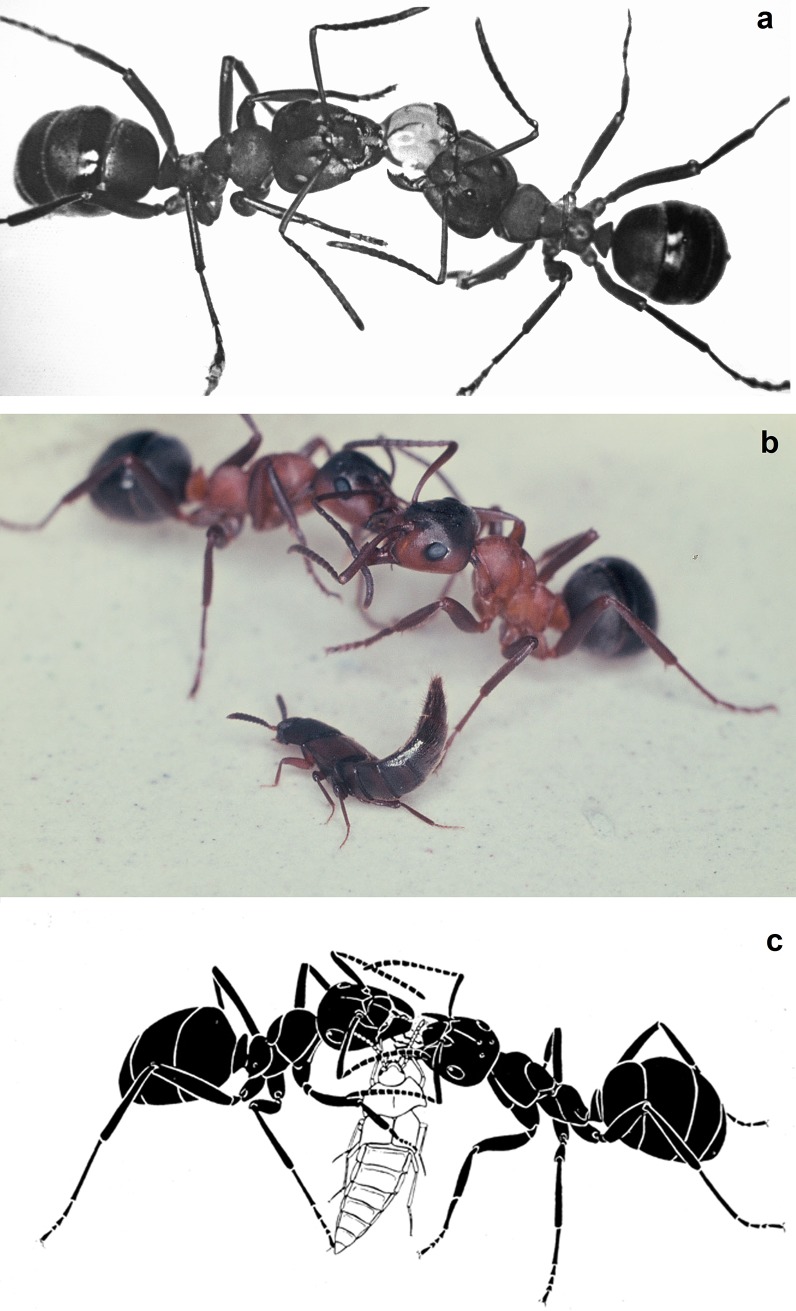
*Dinarda dentata* beetles snatching regurgitated food from the host ants *Formica sanguinea*. a) An ant forager returns to the nest with a full crop and offers food to nest mates. A large, regurgitated food droplet appears between the gaping mandibles of the donor ant. b) Beetles seek out food exchanging ants. c) They insert themselves between the food-exchanging ants and attempt to snatch some of the regurgitated liquid. (Illustration by Turid Hölldobler-Forsyth).

**Fig 13:**
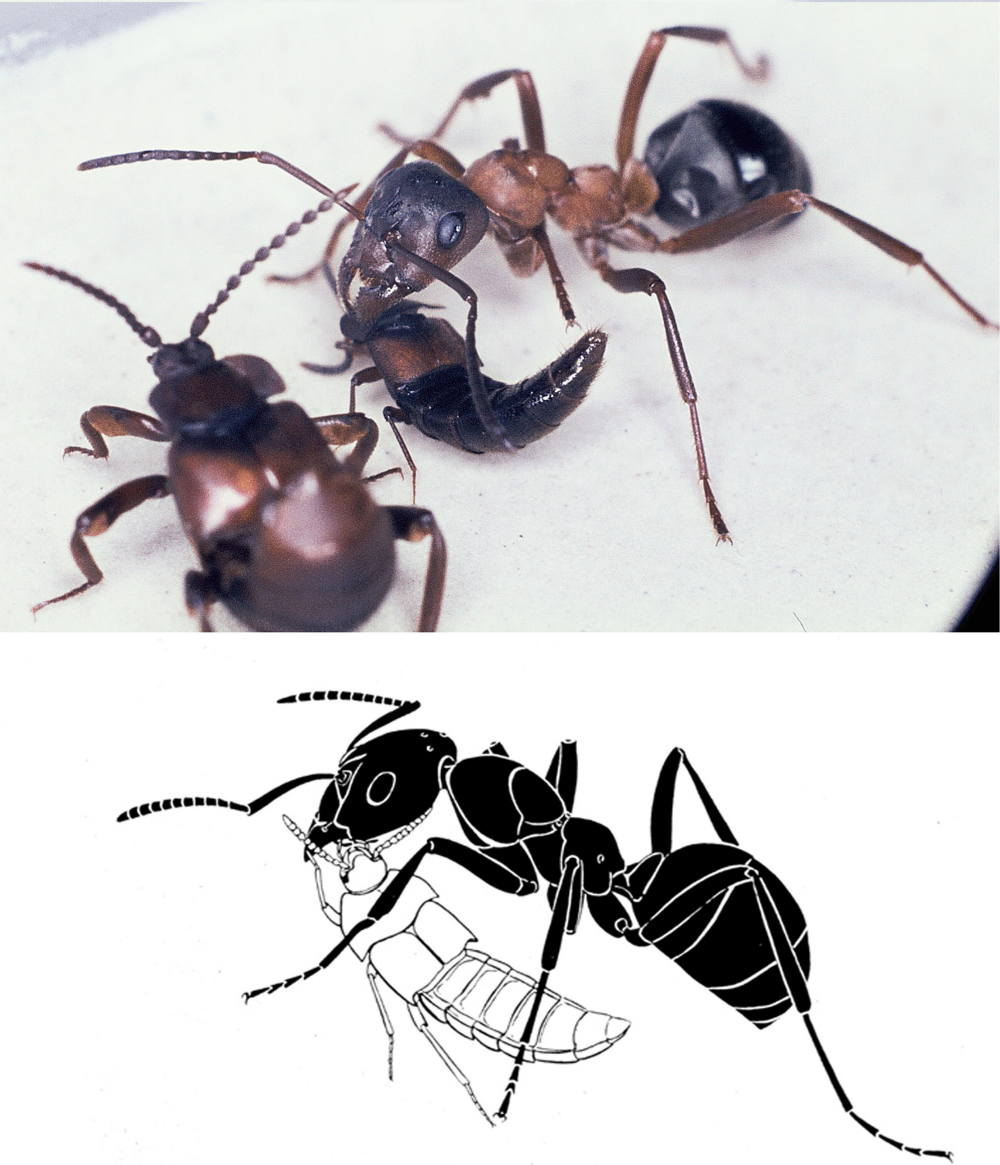
*Dinarda dentata* beetle snatching regurgitated food from host ants. Above: The beetle sneaks underneath of a food-laden ant and stimulates the lower lip (labium) of the ant (below). This sometimes elicits regurgitation of crop contents by the ant (Illustration by Turid Hölldobler-Forsyth).

Foragers that enter the nest with a full crop seek to deliver the collected liquid to nest mates, and occasionally one can see a large regurgitated droplet between the gaping mandibles (Fig 12A). *Dinarda* beetles tend to sneak between the ants engaged in trophallaxis, and snatch a share of their regurgitated food (Fig 13B and 13C). Similar behavior previously reported by Wasmann for *D*. *hagensii* [6] and Parmentier et al [14] provided experimental evidence for trophallaxis in *Dinarda maerkelii*. On other occasions, the beetle may surreptitiously approach a food-laden forager and, by touching the ant’s labium, induce the regurgitation of a small droplet (Fig 13). Shortly afterwards the ant behaves aggressively, but the beetle staves off the attack by raising its abdomen, presenting its appeasement glands secretion [21]. This food exchange behavior between ant and beetle is almost impossible to document photographically. We observed it infrequently inside the nest chambers that were covered by a transparent, red acetate. The illustrations in Figs 12 and 13 by Turid Hölldobler, are based on these observations.

The observational evidence of food exchange was confirmed by the application of the radioactive tracer technique. In Series-A, each group contained 10 ants fed with labeled honey-water and 5 *Dinarda* beetles. In series-B, groups contained 5 previously starved ants that were fed with labeled honey water and then placed with 10 nest mates and 5 *Dinarda* beetles. It was obvious that in series-B trophallaxis was considerably more frequent than in series-A (Table 1). The ants with a fully laden crop offered food droplets to their nestmates. This was mirrored by the larger relative amount of labeled crop contents received by *Dinarda* beetles in the groups from series-B. Impulse counts of beetles were significantly higher than the null/background levels in 40% of beetles in series-B, compared to 20% of beetles in series-A (one-sample Wilcoxon signed rank tests, all p < 0.01). On average, the beetles obtained significantly more food in series-B than in series-A, despite the fact that there were more ant recipients in B (Mann–Whitney U = 75.5, n_A_ = 15, n_B_ = 20, P = 0.014). Although beetles were much smaller than their host ants, those in series B received 32% as much food as host workers. This is likely because trophallaxis acts were more frequent in B, and beetles had more chances to snatch food droplets from the ants. However, it has to be emphasized, the individual *Dinarda* beetles differed greatly in successfully snatching regurgitated food from the ants (Table 2).

**Table 2:** Feeding experiment design and data.

Group Identity	Average group impulse count	Number fed	Sample size		
			Groups	Individuals	Total
**Series A background radiation reads**	26 (SD 4)	NA	—	—	7
**Series A all ants**	988 (SD 402)	30/30	3	10	30
**Series A beetles**	48 (SD 29)	3/15	3	5	15
**Series B background radiation reads**	22 (SD 6)	NA	—	—	8
**Series B all ants**	708 (SD 451)	57/60	4	15	60
**Series B pre-fed ants removed**	325 (SD 64)	37/40	4	10	40
**Series B beetles**	104 (SD 34)	8/20	4	5	20

## Discussion

The genus *Dinarda* belongs to the tribe Oxypodini (subtribe Dinardina) whose known species are all obligate myrmecophiles, associated with specific ant species [13]. *Dinarda dentata* Gravenhorst, 1806 live primarily in nests of *Formica sanguinea*, *D*. *maerkelii* Kiesenwetter, 1842 is associated with *Formica rufa*; *D*. *hagensii* Wassmann, 1889 lives with *Formica exscecta*; *D*. *pygmaea* Wassmann, 1889 lives with *Formica rufibarbis*; and *D*. *lompei* Assing, 2002 has been found with *Formica gagates*. Wheeler [22] lists an additional *Dinarda* species, *D*. *nigrita* found in nests of *Aphaenogaster* sp., but this species has been moved to the genus *Chitosa* where it is the only species in that genus (Alfred Newton, personal communication; see also[23]). The genus *Dinarda* is widely distributed in Europe, certain areas in Western Siberia, Caucasus, and Asia Minor [5, 24]. Bernhauer [25] describes one additional *Dinarda* species, *D*. *africana* from Zanzibar. The description is based on one specimen and no host record is given. The identification and species assignment has not been confirmed. If this assignment is correct it would be the only *Dinarda* species described from another continent than the described European-Asian distributions.

In general, *Dinarda* appears to be quite host specific. *Dinarda dentata* occurs mostly in *Formica sanguinea* nests as Wassmann [6, 13] emphatically states. Nevertheless, he concedes that occasionally this species can be found in the nests of other *Formica* species. In fact, in addition to *F*. *sanguinea* Päivinen [2] lists *F*. *fusca*, *F*. *rufibarbis*, *F*. *exsecta*, *F*. *cinerea* and *F*. *aquilonia*, as hosts Wasmann [13]called these additional species “abnormal” hosts, with *F*. *sanguinea* as the normal host. Indeed, we found *D*. *dentata* only in *F*. *sanguinea* nests. In general, it is fair to say that *Dinarda* are not strictly host specific, but certain species appear to be preferred by the various *Dinarda* species.

Wasmann, and subsequently Donisthorpe [26]were the first to provide observations of the myrmecophilous behavior of *Dinarda dentata* and other *Dinarda* species. Wasmann recognized that this genus represents an intermediate evolutionary grade between the myrmecophilous predators, such as the genus *Pella*, and the brood nest myrmecophilous parasites, such as *Lomechusa* and *Lomechusoides*. He did not see these various degrees of adaptation to particular ecological niches in ant nests and colonies, but argued that *Dinarda* was still in the evolutionary process of reaching the highest level of myrmecophily. On the other hand, he also argued that the host ants evolved specific symphilic instincts for particular myrmecophilous species. For example, he argued the *F*. *sanguinea* possesses a specific symphilic instinct for tolerating *Dinarda dentata*, but not *Dinarda maerkelii*. He explains that when he introduced *D*. *maerkelii* into a *F*. *sanguinea* colony containing *D*. *dentata*, *D*. *maerkelii* was attacked by *F*. *sanguinea*. While Wasmann’s observations of the behavior appear to be accurate, his interpretation has long been criticized and multiple times refuted (see Hölldobler et al.[9]). For instance, he would have made similar observations if he had transplanted *D*. *dentata* specimens between two different *F*. *sanguinea* colonies. Apparently, the introduced, foreign *D*. *dentata* beetles need some time to adjust their “odor bouquet” to that of the host ant colony. After the pioneering discovery by Howard, McDaniel and Blomquist [27] of cuticular hydrocarbon mimicry in the termitophile stapylinid beetle *Trichopsenius frosti* and the subsequent discovery by Vander Meer and Wojcik [28] of the acquisition of the cuticular hydrocarbons by the beetle *Martineziana dutertrei* from its host ants *Solenopsis richteri*, extensive work has been published on chemical mimicry and camouflage in social insect colonies [29–34]. However Parmentier et al. [35] found that in *Dinarda maerkelii*, the cuticular chemical profile deviates significantly from that of the host ant species. The authors suggest that even if the beetles do not mimic the cuticle chemical profiles of their hosts, they can have some other chemical tactics such as compounds that lower aggression in their hosts. This suggestion matches exactly what Hölldobler [21] has proposed is the function of the appeasement secretion. In any case, the behavior of field collected beetles introduced to our laboratory colonies strongly suggests that beetles need a certain “incubation time” before they are largely ignored by the ants. Clearly, it is not the host species that selects and adopts the myrmecophiles, instead the *Dinarda* of a particular species has evolved a preference for a particular host ant species. Therefore, we have to ask what cues might lead *Dinarda dentata* to prefer *F*. *sanguinea*. Although we have not investigated this particular question, some anecdotal observations may suggest an answer. Wheeler [22] described observations in the field of *F*. *sanguinea* colonies moving to a new nest. *Dinarda dentata* beetles were seen on the emigration trail in the company of their host ants. One of us (B.H.), initiated nest emigration in a laboratory nest by making the ants move from one arena via a cardboard bridge to another nest arena. *Dinarda* beetles were observed moving over the bridge during the latter section of the emigration process. It is suggestive that that beetles are able to read the ants trail pheromone, which most likely contains species-specific and even colony-specific components (see [36]). In fact, myrmecophiles that follow their host ants’ trails are not only known from species, which live in colonies of legionary ants, but also from myrmecophiles associated with stationary ant nests. For example, the staphylinid beetle *Myrmecosaurus ferrugineus* follows fire ant odor trails [37], or the myrmecophilous cricket *Myrmecophilus* follow trails of *Formica obscuripes* [38], and species of the myrmecophilous cockroach *Attaphila* follow trails of their respective leafcutter ant host species [1, 39].

Our histological investigations have not revealed a trace of preadaptation of the so-called adoption glands that enable rove beetles *Lomechusa* and *Lomechusoides* to be adopted into the brood chambers of their host ants, where beetles unopposed prey on the ants’ brood and are even fed by the ants [8, 9]. However, *Dinarda* beetles are equally well endowed with exocrine glands at the tip of the abdomen and our observational evidence suggests that some of the glands or all of them are involved in the chemical appeasement process, which is a “gentle” chemical defense. The ants are attracted to the secretion of this appeasement gland complex; they lick the abdominal tip and this gives the beetle enough time to escape a possible attack. We were able to confirm previous observations that the beetles may insert themselves between two ants engaged in trophallaxis [1, 6], and by employing radioactive tracer experiments we demonstrated that some beetles were quite successful in obtaining considerable food quantities from the host ants, while others did not obtain food in our experiments. Clearly, the main nutrition of *Dinarda dentata* is obtained by scavenging the middens of their host ants.

In conclusion, we can reiterate that our histological studies and behavioral analyses strongly support the hypothesis that *Dinarda* represent a grade between the scavenger-predator mode of myrmecophily and the brood nest parasites.
